# RNA‐sequencing of WFS1‐deficient pancreatic islets

**DOI:** 10.14814/phy2.12750

**Published:** 2016-04-06

**Authors:** Marilin Ivask, Alison Hugill, Sulev Kõks

**Affiliations:** ^1^ Department of PathophysiologyInstitute of Biomedicine and Translational MedicineUniversity of TartuTartuEstonia; ^2^ Mammalian Genetics UnitMedical Research CouncilHarwellOxfordshireUnited Kingdom

**Keywords:** Insulin, RNA‐sequencing, Trpm5, Wfs1

## Abstract

Wolfram syndrome, an autosomal recessive disorder characterized by juvenile‐onset diabetes mellitus and optic atrophy, is caused by mutations in the *WFS1* gene. *WFS1* encodes an endoplasmic reticulum resident transmembrane protein. The *Wfs1*‐null mice exhibit progressive insulin deficiency and diabetes. The aim of this study was to describe the insulin secretion and transcriptome of pancreatic islets in WFS1‐deficient mice. WFS1‐deficient (Wfs1KO) mice had considerably less pancreatic islets than heterozygous (Wfs1HZ) or wild‐type (WT) mice. Wfs1KO pancreatic islets secreted less insulin after incubation in 2 and 10 mmol/L glucose and with tolbutamide solution compared to WT and Wfs1HZ islets, but not after stimulation with 20 mmol/L glucose. Differences in proinsulin amount were not statistically significant although there was a trend that Wfs1KO had an increased level of proinsulin. After incubation in 2 mmol/L glucose solution the proinsulin/insulin ratio in Wfs1KO was significantly higher than that of WT and Wfs1HZ. RNA‐seq from pancreatic islets found melastatin‐related transient receptor potential subfamily member 5 protein gene (*Trpm5*) to be downregulated in WFS1‐deficient mice. Functional annotation of RNA sequencing results showed that WFS1 deficiency influenced significantly the pathways related to tissue morphology, endocrine system development and function, molecular transport network.

## Introduction

Wolfram syndrome (WS, OMIM 222300) is a rare autosomal recessive neurodegenerative disorder caused by mutations in the wolframin gene (*WFS1*). The main symptoms of the disease are juvenile‐onset diabetes mellitus, progressive optic atrophy, diabetes insipidus and deafness (Barrett and Bundey 1997; Inoue et al. 1998; Strom et al. 1998). Nonautoimmune degeneration of pancreatic *β*‐cells is common for WS patients (Karasik et al. 1989). In patients with WS high levels of endoplasmic reticulum (ER) stress and pancreatic *β*‐cell death may be associated with impaired *β*‐cell function as in the case of type 2 diabetes (T2D) (Fonseca et al. 2005). Genetic studies have also shown a link between the risk of developing T2D and *WFS1* (Minton et al. 2002; Sandhu et al. 2007; Florez et al. 2008; Franks et al. 2008; van Hoek et al. 2008; Cheurfa et al. 2011).

WFS1 is a 100 kDa glycoprotein composed of 890 amino acids and has nine transmembrane segments. Inside the cell WFS1 localizes in the ER (Takeda et al. 2001; Hofmann et al. 2003; Philbrook et al. 2005). Its location in the ER suggests that WFS1 could participate in membrane trafficking, processing proteins and/or regulation of the Ca^2+^ homeostasis in the ER (Takeda et al. 2001). WFS1 itself may be a Ca^2+^‐channel in the ER or its regulator (Osman et al. 2003). WFS1 is expressed at highest levels in brain, heart and pancreatic *β*‐cells (Inoue et al. 1998; Strom et al. 1998; Hofmann et al. 2003; Ishihara et al. 2004).

*Wfs1*‐mutant mice have been generated in several laboratories (Ishihara et al. 2004; Riggs et al. 2005; Luuk et al. 2008; Koks et al. 2009). One model is a knock‐out of the second exon in [(129Sv × B6) × B6] F2 background and the resulting WFS1 protein lacks first 183 amino acids and NH_2_‐terminus (Ishihara et al. 2004). Riggs et al. (2005) generated a pancreatic islet specific conditional *Wfs1* mouse model disrupting exon 8. The WFS1‐deficient mice (Wfs1^tm1Koks^) used in current study have also the exon 8 disrupted resulting in the deletion of amino acids 360–890 (Luuk et al. 2008; Koks et al. 2009).

Mice with a loss of function of the *Wfs1* gene exhibit *β*‐cell loss, which may result from high levels of ER stress, cell cycle dysfunction, and apoptosis (Fonseca et al. 2005; Riggs et al. 2005; Takei et al. 2006; Yamada et al. 2006). Insulin secretion from isolated islets of mice with a disrupted *Wfs1* gene has been shown to be impaired (Ishihara et al. 2004). However, there is no more detailed analysis of insulin secretion in *Wfs1*‐mutant mice.

RNA‐sequencing (RNA‐seq) has become a great tool for transcriptomic studies without the need of previous knowledge of the targets, allowing identifying novel transcripts and gene expression changes due to some disease (Mortazavi et al. 2008). There have been studies regarding pancreatic islets' transcriptome and differential gene expression caused by diabetes (Eizirik et al. 2012; Ku et al. 2012; Moran et al. 2012). However, the transcriptional changes in the islets induced by the deletion of *WFS1* are not known.

The aim of this study was to perform analysis of insulin secretion and proinsulin content in isolated pancreatic islets of WFS1‐deficient mice (Wfs1^tm1Koks^) (Luuk et al. 2008; Koks et al. 2009). The transcriptome of islets was analyzed using RNA‐seq to describe transcriptional changes in pancreatic islets associated with *Wfs1* deficiency and potentially related to the changes in insulin secretion.

## Methods

### Animals

The animal experiments described in this study were performed with permission from Estonian National Board of Animal Experiments (No. 71, April 8th, 2011) and in accordance with the European Communities Directive (86/609/EEC).

Generation of *Wfs1* mutant (Wfs1KO, Wfs1^tm1Koks^) mice has been described elsewhere (Luuk et al. 2008; Koks et al. 2009). Briefly, most of exon 8 was replaced with a LacZ cassette resulting in the deletion of amino acids 360–890 in the *WFS1* protein. The expressed truncated protein is a fusion between *WFS1* residues 1–359 (N‐terminal part) and LacZ lacking at least seven of the nine transmembrane domains and the C‐terminal portion of endogenous *WFS1* protein (Luuk et al. 2008; Koks et al. 2009). In the study 3 genotypes of mice were used: wild‐type (WT), heterozygotes for *Wfs1* mutation (Wfs1HZ) and homozygotes for *Wfs1* mutation (Wfs1KO). All studies were performed on male F2 hybrids (129S6/SvEvTac × C57BL/6) and mice were 5–6‐month‐old at the time of the experiment. Mice were housed in groups of 6–8 at 20 ± 2°C under 12‐h/12‐h light/dark cycle with free access to food and water.

### Isolation of pancreatic islets

Pancreatic islets were isolated as previously described (Shimomura et al. 2009) from six mice in each genotype. Briefly, mice were executed by cervical dislocation and the islets isolated by collagenase Type XI digestion (Sigma‐Aldrich, St. Louis, MO, USA, final concentration 1 mg/mL). The inflated pancreas was dissected out and incubated in a 37°C water bath for 13 min. Only pancreata inflated 100% were used. The tissue was washed twice with 0.2% BSA (Sigma) and HBSS (Sigma) solution. The islets were handpicked under stereomicroscope in 0.2% BSA and HBSS solution. Finally, the islets were picked into high glucose (4.5 g/L) DMEM/Ham's F12 media (PAA/GE Healthcare Life Sciences, Chicago, IL, USA), containing 10% FBS (Gibco/Thermo Fisher Scientific Inc., Waltham, MA, USA), 100 U/mL penicillin and 100 *μ*g/mL streptomycin (Gibco) and 20 mmol/L l‐glutamine (Invitrogen/Thermo Fisher Scientific Inc., Waltham, MA, USA). Islets were incubated overnight at 37°C before insulin secretion assay. During the picking the number of islets was also counted manually.

### Insulin secretion assay

Islets were incubated for 1 h at 37°C and 5% CO_2_ in 0.2% BSA and Krebs‐Ringer solution (KRBH, 140 mmol/L NaCl, 0.5 mmol/L NaH_2_PO_4_, 2 mmol/L NaHCO_3_, 3.6 mmol/L KCl, 0.5 mmol/L MgSO_4_, 2.6 mmol/L CaCl_2_·2H_2_O, 5 mmol/L HEPES, pH 7.4) containing 2 mmol/L glucose. Then, islets were incubated for 1 h at 37°C and 5% CO_2_ in selected assay solution (KRBH and 2 mmol/L, 10 mmol/L or 20 mmol/L glucose or 200 *μ*mol/L tolbutamide and 2 mmol/L glucose). Tolbutamide (Sigma) was first dissolved in 0.2% DMSO (Sigma) with final concentration of 200 *μ*mol/L. Each assay media group contained 5 islets and was in duplicate per genotype. After incubation the supernatant was collected and stored at −20°C until ELISA analysis. To determine total insulin content, insulin was extracted from the same islets using 95:5 ethanol:acetic acid solution (Shimomura et al. 2009).

Insulin concentration was determined with Ultra Sensitive Mouse Insulin ELISA Kit (Crystal Chem Inc., Downers Grove, IL, USA). Insulin amount was determined separately from secretion and islet content samples. To reduce the effect of variation in islet size on insulin secretion, the secreted amount of insulin was normalized to the content of insulin (secreted insulin divided by insulin content).

Proinsulin concentration was measured from islet content samples with Mouse Proinsulin ELISA Kit (Wuhan EIAab Science Co., Ltd., Wuhan, China). Proinsulin was also determined from secretion samples, but the results were too low and did not reach the detection range. Differences in the amount of proinsulin were normalized to the number of islets used in each well. To compare proinsulin/insulin ratio between genotypes the proinsulin concentration was divided with corresponding normalized insulin.

### Transcriptome analysis

RNA‐sequencing was used for transcriptome analysis. Pancreatic islets from 4 animals were used in each genotype group. Total RNA was isolated from islets using the RNeasy Mini Kit (Qiagen Inc., Valencia, CA, USA) according to manufacturer's protocol and cDNA for sequencing was synthesized using Ovation RNA‐Seq System V2 (NuGEN Technologies Inc., San Carlos, CA, USA), 10 ng of total RNA was used. SOLiD DNA Fragment library kit (cDNA input 2 *μ*g) was used to generate libraries and quality was controlled with the Agilent Bioanalyzer 2100 (Agilent Technologies Inc., Santa Clara, CA, USA) before sequencing. The libraries were marked with different barcodes and pooled together for the template preparation with automated SOLiD EZ Bead E80 System and its consumables (Life Technologies/Thermo Fisher Scientific Inc., Waltham, MA, USA). The SOLiD 5500xl System and paired end (75 bp forward and 35 bp reverse) chemistry (Life Technologies) were used for sequencing. Samples from each animal were sequenced and analyzed separately.

All the raw sequences are deposited in the sequence read archive (http://trace.ncbi.nlm.nih.gov/Traces/sra/) under the accession number GSE65929.

### Quantitative real‐time PCR validation

The confirmatory quantitative real‐time PCR (RT‐PCR) was performed using TaqMan gene expression assays and chemistry (Life Technologies). Samples were treated with TURBO DNA‐free kit (Ambion/Thermo Fisher Scientific Inc., Waltham, MA, USA), according to the manufacturer's instructions to remove contaminating genomic DNA. Total RNA of 10 ng from each sample was subjected to cDNA synthesis using High Capacity cDNA Reverse Transcription Kit (Life Technologies) following the manufacturer's protocol. The expression of *Glipr2* (glioma pathogenesis‐related protein 2, Mm01341451_m1, FAM), *Trpm5* (Mm01129032_m1, FAM), *Gad1* (glutamate decarboxylase 1, Mm00725661_s1, FAM), *MaoB* (monoamine oxidase B, Mm00555412_m1, FAM), *ApoE* (apolipoprotein E, Mm01307193_g1, FAM) was analyzed using the ABI Prism 7900 HT Sequence Detection System (Life Technologies). The same samples were analyzed two times, but three times for *Trpm5*.

### Statistical analysis

Islet and insulin secretion data are presented as mean ± SEM. Data were analyzed using either one‐way or two‐way ANOVA, followed by Tukey post test. A *P* value of <0.05 was considered statistically significant (*P *<* *0.05). The statistical analysis was performed using GraphPad Prism 5 software (GraphPad Software Inc., La Jolla, CA, USA).

Data from RT‐PCR are presented as mean of 2^−ΔCt^ ± SEM calculated in relation to the TaqMan Endogenous Control assay for *Hprt* (hypoxanthine phosphoribosyltransferase, Mm00446968_m1, VIC, primer limited). Data for studied genes were analyzed by one‐way ANOVA and Tukey post hoc test using GraphPad Prism 5 software (GraphPad Software Inc.) and a *P* value <0.05 was considered significant.

For RNA‐seq data analysis sequencing reads were mapped to the mouse genome (version mm10) using the genomic analysis software LifeScope (Life Technologies). Data were further analyzed for the differential expression with the edgeR package implemented in the statistical software R (http://www.r-project.org/).

### Functional annotation of transcriptome

Ingenuity Pathway Analysis (IPA, Ingenuity Systems, http://www.ingenuity.com) was used to define the functional networks of differentially expressed genes. The uploaded dataset contained gene identifiers, corresponding false discovery rate (FDR) and fold change values.

As standard workflow in IPA each gene identifier was mapped to its corresponding gene object in the Ingenuity Pathways Knowledge Base to generate the list of focus genes. Based on the connectivity of these focus genes the IPA software generates networks and calculates a significance score for each network. The score is generated from the *P* value and is displayed as the negative logarithm of that *P* value. This score indicates the likelihood that the assembly of a set of focus genes in a network could be explained by random chance alone. A score of 2 indicates that there is a 1 in 100 chance that the focus genes are together in a network due to random chance.

Functional analysis was performed on the entire dataset and filtered to get only FDR corrected significant genes. Statistical significance filtering was used to increase the focus and specificity of analysis.

## Results

### The number of pancreatic islets

**Figure 1:**
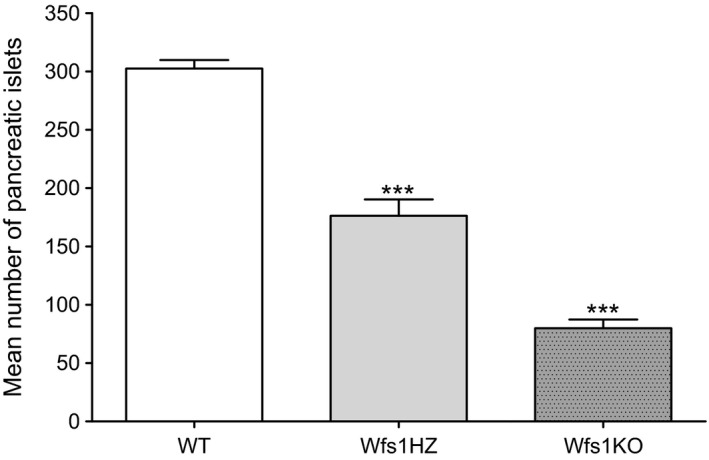
Comparison of the number of isolated pancreatic islets between genotypes. The difference in the number of isolated pancreatic islets per pancreas between genotypes was statistically highly significant (***P <* *0.001) by one‐way ANOVA. Data plotted as mean ± SEM,* n* = 6.

The number of pancreatic islets was manually counted during handpicking. The difference in number of isolated pancreatic islets between genotypes was statistically highly significant (WT 303 ± 7.3, Wfs1HZ 176 ± 14 and Wfs1KO 80 ± 7.5, *P *<* *0.001) (Fig. 1). Wfs1KO animals had remarkably less pancreatic islets than WT or Wfs1HZ animals indicating a genotypic effect.

### Insulin secretion

**Figure 2:**
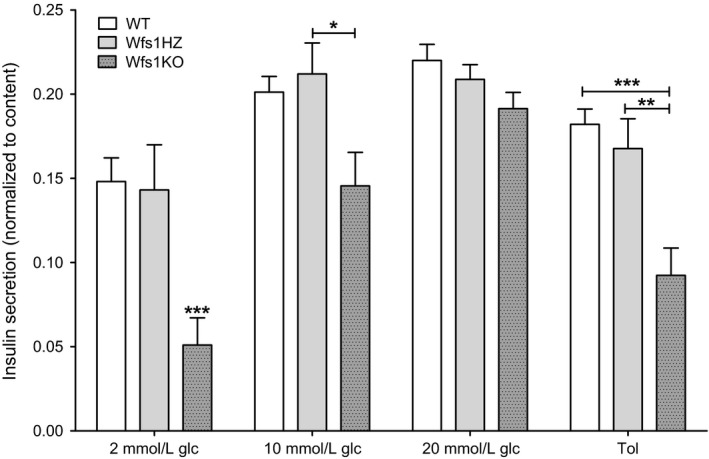
Insulin secretion from isolated islets. Insulin secretion from isolated islets of WT, Wfs1HZ and Wfs1KO littermates are compared in response to 2 mmol/L, 10 mmol/L or 20 mmol/L glucose (glc) or 200 *μ*mol/L tolbutamide (Tol) solution and normalized to total insulin content. Insulin secretion from Wfs1KO islets was decreased after incubation in 2 mmol/L and 10 mmol/L glucose and tolbutamide solution, but not after stimulation with 20 mmol/L glucose solution. Statistical analysis by two‐way ANOVA, where *P <* *0.05, **P <* *0.01 and ***P <* *0.001. Data plotted as mean ± SEM, *n* = 6.

The basal insulin secretion after incubation in 2 mmol/L glucose (Fig. 2) for Wfs1KO islets (0.051 ± 0.016) was reduced compared to WT (0.148 ± 0.014) and Wfs1HZ (0.143 ± 0.027) islets (*P *<* *0.001).

When islets were stimulated with 10 mmol/L glucose (Fig. 2) solution, the difference in secreted insulin between WT (0.201 ± 0.009) and Wfs1KO (0.146 ± 0.020) was significant (*P *<* *0.05), the difference between Wfs1HZ (0.212 ± 0.018) and Wfs1KO was highly significant (*P *<* *0.01).

However, after stimulation with 20 mmol/L glucose (Fig. 2) there was no significant difference in stimulation of insulin secretion between the genotypes (WT 0.220 ± 0.0095, Wfs1HZ 0.209 ± 0.0088 and Wfs1KO 0.191 ± 0.0096, *P *>* *0.05).

The response to the sulfonylurea tolbutamide (Fig. 2) was also significantly impaired in Wfs1KO islets (0.092 ± 0.016) compared to WT (0.182 ± 0.009, *P *<* *0.001) and Wfs1HZ (0.168 ± 0.018, *P *<* *0.01) islets.

The dose‐dependent‐stimulating effect of glucose is seen in all the genotypes, although insulin secretion did not statistically differ after stimulation with 10 mmol/L and 20 mmol/L glucose. The lower normalized insulin amount seen in Wfs1KO mice was primarily due to decreased insulin secretion, because the insulin content in pancreatic islets did not significantly differ between genotypes.

### Proinsulin amount and proinsulin/insulin ratio

**Figure 3:**
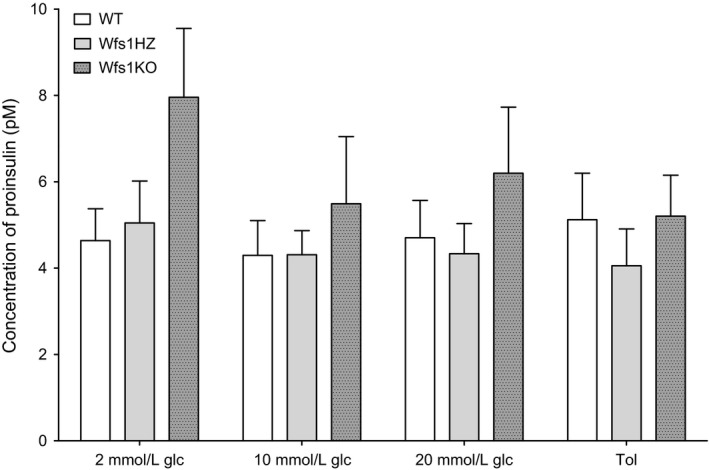
Comparison of the average amount of proinsulin per islet between the genotypes. The proinsulin amount per islet was not significantly different between the genotypes (*P *>* *0.05) after stimulation with 200 *μ*mol/L tolbutamide (Tol) or various glucose (glc) solutions. Statistical analysis by two‐way ANOVA, where *P <* *0.05, **P <* *0.01 and ***P <* *0.001. Data plotted as mean ± SEM,* n* = 6.

The average proinsulin amount per islet (Fig. 3) was not significantly different between the genotypes (2 mmol/L glucose: WT 4.636 ± 0.739 pmol/L, Wfs1HZ 5.047 ± 0.969 pmol/L and Wfs1KO 7.958 ± 1.59 pmol/L; 10 mmol/L glucose: WT 4.296 ± 0.805 pmol/L, Wfs1HZ 4.309 ± 0.558 pmol/L and Wfs1KO 5.489 ± 1.56 pmol/L; 20 mmol/L glucose: WT 4.703 ± 0.864 pmol/L, Wfs1HZ 4.333 ± 0.700 pmol/L and Wfs1KO 6.199 ± 1.53 pmol/L; tolbutamide: WT 5.120 ± 1.08 pmol/L, Wfs1HZ 4.055 ± 0.852 pmol/L and Wfs1KO 5.202 ± 0.950 pmol/L; *P *>* *0.05).

**Figure 4:**
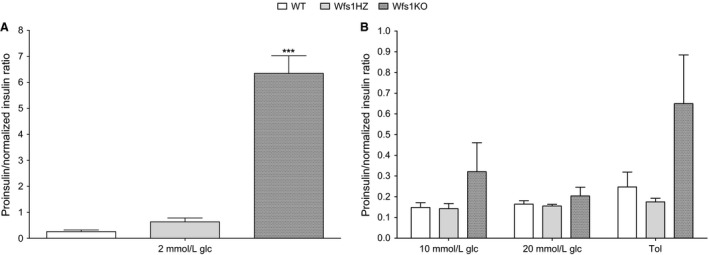
Proinsulin to insulin ratio. (A) The higher Wfs1KO proinsulin/insulin ratio was significantly different from WT and Wfs1HZ (***P <* *0.001) at 2 mmol/L glucose (glc). (B) The differences between the genotypes in proinsulin/insulin ratio at higher glucose and tolbutamide (Tol) solutions was not significant (*P *>* *0.05). Statistical analysis by two‐way ANOVA, where *P <* *0.05, **P <* *0.01 and ***P <* *0.001. Data plotted as mean ± SEM,* n* = 6.

When islets were incubated in 2 mmol/L glucose solution, there was no significant difference in proinsulin/insulin ratio between WT (0.256 ± 0.065) and Wfs1HZ (0.632 ± 0.150, *P *>* *0.05). However, the much higher Wfs1KO proinsulin/insulin ratio (6.345 ± 0.680) was extremely different from ratios of WT and Wfs1HZ genotypes (*P *<* *0.001) (Fig. 4A). After treating islets with other glucose doses (10 mmol/L glucose: WT 0.148 ± 0.023, Wfs1HZ 0.143 ± 0.025 and Wfs1KO 0.321 ± 0.140; 20 mmol/L glucose: WT 0.165 ± 0.016, Wfs1HZ 0.156 ± 0.008 and Wfs1KO 0.204 ± 0.042) and tolbutamide (WT 0.247 ± 0.072, Wfs1HZ 0.175 ± 0.0174 and Wfs1KO 0.650 ± 0.235) there were no significant difference in proinsulin/insulin ratio between the genotypes (*P *>* *0.05) (Fig. 4B). However, the ratio of proinsulin/insulin was constantly higher in Wfs1KO mice indicating a larger amount of unprocessed insulin in the islets.

### Transcriptome analysis

The number of reads per sample varied from 27 million to 72 million. The number of transcripts mapped to mouse genome was 22613 between Wfs1KO and WT, 22619 between Wfs1KO and Wfs1HZ and 22591 between Wfs1HZ and WT. EdgeR exact test of RNA‐seq data revealed 20 genes with FDR under 0.05 (FDR < 0.05) between Wfs1KO and WT islets (Table 1A), between Wfs1KO and Wfs1HZ were 13 genes with FDR < 0.05 (Table 1B). With FDR < 0.2 there were additional 29 genes different between Wfs1KO and WT (data not shown), 7 genes between Wfs1KO and Wfs1HZ (data not shown) and one gene between Wfs1HZ and WT (*Dpep1*, logFC = 3.507, logCPM = 2.759, *P *=* *7.21E–06, FDR = 0.173). The most significantly downregulated gene associated with insulin secretion and diabetes in WFS1‐deficient islets following *Wfs1* was melastatin‐related transient receptor potential subfamily member 5 (*Trpm5*).

**Table 1:** RNA‐seq results of pancreatic islets showing genes with FDR < 0.05

Gene	Log ratio	*P* value	False discovery rate (*q*‐value)	Entrez gene name
(A). Wfs1KO compared to WT				
Wfs1	−2.663	2.18E–12	5.24E–08	Wolfram syndrome 1 (wolframin)
Glipr2	3.139	1.42E–09	1.71E–05	GLI pathogenesis‐related 2
Trpm5	−2.422	4.05E–09	3.25E–05	Transient receptor potential cation channel, subfamily M, member 5
Gad1	−2.671	9.94E–08	5.98E–04	Glutamate decarboxylase 1 (brain, 67 kDa)
Spock1	−1.801	8.71E–07	4.19E–03	Sparc/osteonectin, cwcv and kazal‐like domains proteoglycan (testican) 1
Sprr1a	2.623	1.36E–06	5.44E–03	Small proline‐rich protein 1A
Bcat1	1.821	1.67E–06	5.75E–03	Branched chain amino‐acid transaminase 1, cytosolic
Csf3	2.332	2.03E–06	6.12E–03	Colony‐stimulating factor 3 (granulocyte)
Nrxn1	−1.404	5.14E–06	1.38E–02	Neurexin 1
Prss23	1.769	7.19E–06	1.62E–02	Protease, serine, 23
Aw551984	−1.551	7.40E–06	1.62E–02	Expressed sequence AW551984
Cxcl9	3.394	8.28E–06	1.66E–02	Chemokine (C‐X‐C motif) ligand 9
MaoB	−1.86	1.02E–05	1.89E–02	Monoamine oxidase B
Kcns3	4.122	1.17E–05	2.02E–02	Potassium voltage‐gated channel, delayed‐rectifier, subfamily S, member 3
Zfp36	1.709	1.71E–05	2.74E–02	ZFP36 ring finger protein
Egr1	1.392	2.65E–05	3.97E–02	Early growth response 1
ApoE	1.712	2.91E–05	3.97E–02	Apolipoprotein E
Itgb3	1.701	2.97E–05	3.97E–02	Integrin, beta 3 (platelet glycoprotein IIIa, antigen CD61)
Ccdc85B	1.566	3.38E–05	4.07E–02	Coiled‐coil domain containing 85B
Cnnm1	−1.767	3.38E–05	4.07E–02	Cyclin M1
(B). Wfs1KO compared to Wfs1HZ				
Trpm5	−3.292	2.04E–12	4.92E–08	Transient receptor potential cation channel, subfamily M, member 5
Gad1	−2.834	6.40E–08	7.70E–04	Glutamate decarboxylase 1 (brain, 67kDa)
Bcat1	1.871	4.53E–07	2.82E–03	Branched chain amino‐acid transaminase 1, cytosolic
Serpina7	2.855	4.68E–07	2.82E–03	Serpin peptidase inhibitor, clade A (alpha‐1 antiproteinase, antitrypsin), member 7
Dpyd	−1.746	2.34E–06	9.38E–03	Dihydropyrimidine dehydrogenase
Spock1	−1.758	3.01E–06	1.01E–02	Sparc/osteonectin, cwcv and kazal‐like domains proteoglycan (testican) 1
Npas4	−3.008	3.35E–06	1.01E–02	Neuronal PAS domain protein 4
Cxcl9	3.563	4.36E–06	1.14E–02	Chemokine (C‐X‐C motif) ligand 9
Sprr1a	2.328	4.73E–06	1.14E–02	Small proline‐rich protein 1A
Insrr	−2.356	7.32E–06	1.49E–02	Insulin receptor‐related receptor
Cpb2	2.129	7.44E–06	1.49E–02	Carboxypeptidase B2 (plasma)
Bhlha15	1.759	2.38E–05	4.40E–02	Basic helix‐loop‐helix family, member a15

FDR, false discovery rate; Wfs1HZ, pancreatic islets of *Wfs1* heterozygous mice; Wfs1KO; pancreatic islets of *Wfs1* deficient mice.

Various ER stress markers from RNA‐seq (Wfs1KO islets compared to WT islets) are presented in Table 2. The FDR and log ratio values of these ER stress markers were not significant.

**Table 2:** RNA‐seq results of ER stress markers (Wfs1KO compared to WT islets)

Gene	Log ratio	*P* value	False discovery rate (q‐value)	Entrez gene name
Atf6	0.535	0.095	1	Activating transcription factor 6
Ern1 (Ire1*α*)	−0.211	0.795	1	Endoplasmic reticulum to nucleus signaling 1 (inositol‐requiring enzyme 1)
Xbp1	−0.017	0.697	1	X‐box‐binding protein 1
Eif2ak3 (Perk)	−0.214	0.409	1	Eukaryotic translation initiation factor 2 *α* kinase 3 (protein kinase R‐like endoplasmic reticulum kinase)
Atf4	0.193	0.633	1	Activating transcription factor 4
Ddit3 (Chop)	0.215	0.595	1	DNA‐damage inducible transcript 3 (C/EBP homologous protein)
Hspa5 (Bip)	0.347	0.176	1	Heat‐shock 70 kDa protein 5 (glucose‐regulated protein, 78 kDa)

ER, endoplasmic reticulum; WT, wild‐type.

**Figure 5:**
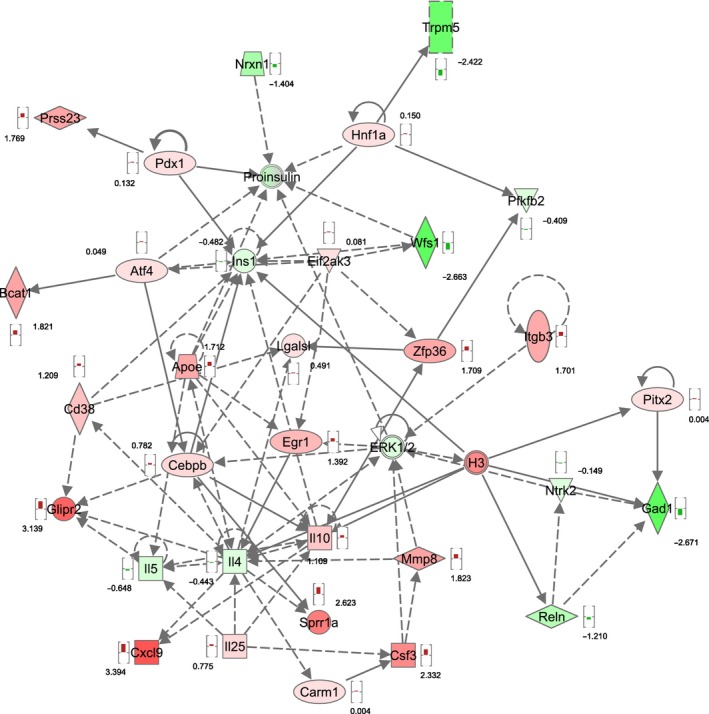
Associated functional network of Wfs1KO compared to WT. Functional annotation revealed that genes with highest expressional changes because of WFS1 deficiency belong to the “tissue morphology, endocrine system development and function, molecular transport” functional network. Red symbols are upregulated genes, green symbols are downregulated genes, and the numbers reflect the *t*‐value of the statistical comparison with Bayesian moderated *t*‐test.

**Figure 6:**
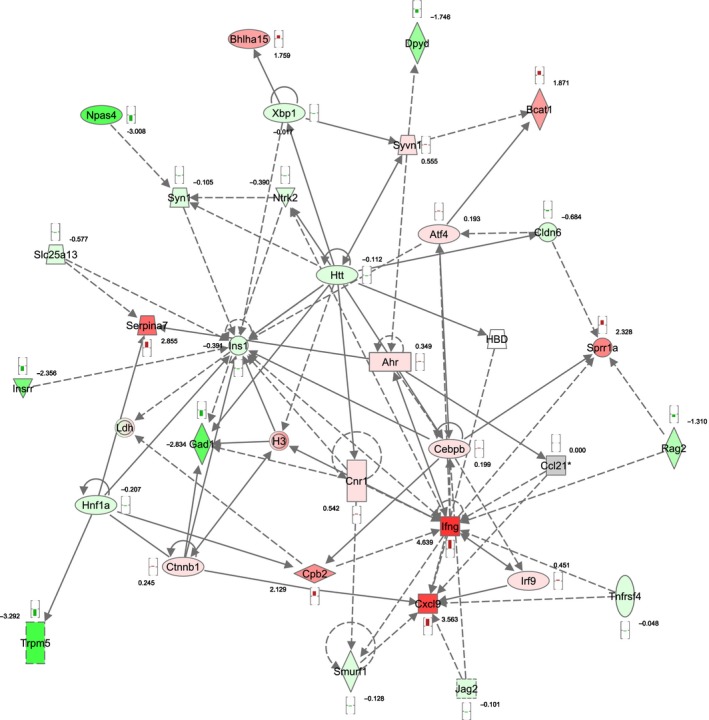
Associated functional network of Wfs1KO compared to Wfs1HZ. Functional annotation revealed that differences in genes with highest expressional changes belong to the “cellular development, cellular growth and proliferation, hepatic system development and function” functional network. Red symbols are upregulated genes, green symbols are downregulated genes, and the numbers reflect the *t*‐value of the statistical comparison with Bayesian moderated *t*‐test.

Ingenuity functional pathway analysis software was used for more general functional annotation of the differential gene sets. Network analysis of the genes with lowest *P* values (filter set to *P *<* *0.05 after FDR correction) of Wfs1KO compared to WT revealed significant enrichment of tissue morphology, endocrine system development and function, molecular transport network (score 33, Fig. 5). Analysis of Wfs1KO compared to Wfs1HZ revealed a network associated with cellular development, cellular growth and proliferation, hepatic system development and function (score 28, Fig. 6).

### Quantitative real‐time PCR confirmation

**Figure 7:**
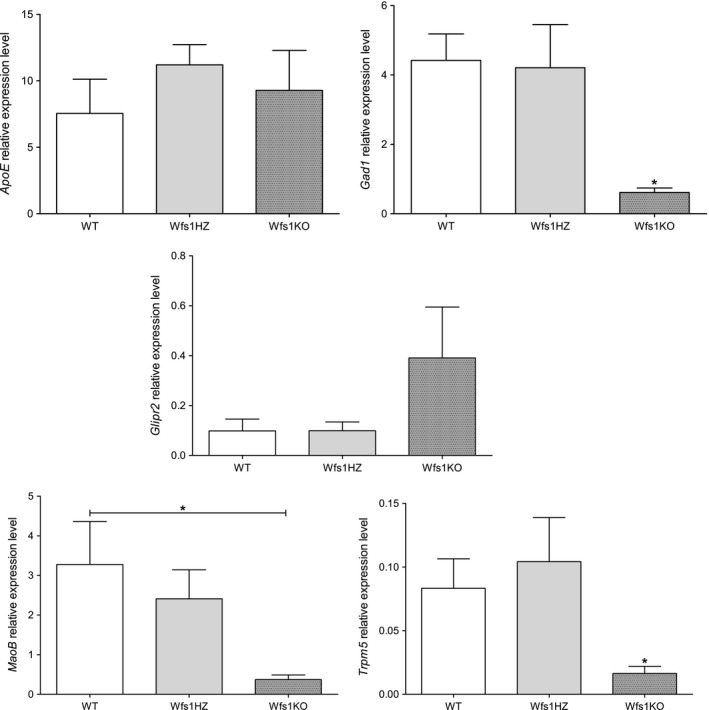
Results of validating RT‐PCR. *ApoE* and *Glipr2* were upregulated according to RNA‐seq, but RT‐PCR did not confirm it statistically, although there was a trend that *ApoE* and *Glipr2* are upregulated in Wfs1KO islets. The downregulation of *Gad1*,* MaoB,* and *Trpm5* was confirmed in Wfs1KO islets. Statistical analysis by one‐way ANOVA followed by Tukey post hoc test, where *P <* *0.05, **P <* *0.01 and ***P <* *0.001. Data plotted as mean ± SEM,* n* = 4.

To determine whether similar changes in gene expression could be observed with RT‐PCR analysis the expression of *Glipr2, Trpm5, Gad1, MaoB, ApoE* genes and the values of three genotypes were compared with one‐way ANOVA followed by Tukey post hoc test (Fig. 7). *ApoE* and *Glipr2* were upregulated according to RNA‐seq, but RT‐PCR did not confirm it statistically, although there was a trend that *ApoE* and *Glipr2* are upregulated in Wfs1KO islets. The downregulation of *Gad1*,* MaoB,* and *Trpm5* was confirmed in Wfs1KO islets (*P *<* *0.05).

## Discussion

Previous studies have shown that WFS1‐deficient (Wfs1^tm1Koks^) mice have disturbed blood glucose regulation and their plasma insulin level is lower, but plasma proinsulin level higher (Luuk et al. 2009; Noormets et al. 2011; Terasmaa et al. 2011). In this study, the difference in transcriptome and insulin secretion from isolated pancreatic islets of WFS1‐deficient (Wfs1^tm1Koks^) mice were studied, also differences in proinsulin levels and proinsulin/insulin ratio were detected.

WFS1‐deficient mice had considerably fewer pancreatic islets than heterozygous or wild‐type mice during isolation. This has also been noticed in previous studies (Ishihara et al. 2004; Riggs et al. 2005). The immunohistochemistry of the same WFS1‐deficient (Wfs1^tm1Koks^) mouse line has been done by Terasmaa et al. (2011) showing that the islets of WFS1‐deficient mice also seem to be smaller. *β*‐cells with defective WFS1 are more prone to ER stress induced apoptosis (Ishihara et al. 2004; Philbrook et al. 2005; Riggs et al. 2005; Yamada et al. 2006) which could cause the reduced number of pancreatic islets in WFS1‐deficient mice (Riggs et al. 2005).

According to current RNA‐seq results the down‐ or upregulation of various ER stress markers was not statically significant, although it has been shown that the levels of ER stress markers from all three UPR pathways are increased in the absence of WFS1 (Yamada et al. 2006; Fonseca et al. 2010). WFS1 is shown to downregulate ATF6*ɑ* and its downstream targets (Fonseca et al. 2010). It has been shown that in the pancreatic *β*‐cells of WFS1‐deficient mice the expression of GRP78 and GRP94 has increased, also levels of spliced XPB1 (Yamada et al. 2006). The possible explanation may be that gene expression might not always correspond to protein level and most of the ER stress marker studies have been done in cell lines and also the mouse line (Wfs1^tm1Koks^) used in this study that is not a complete knock‐out of *Wfs1*. A truncated *WFS1* protein lacking amino acids 360–890 (C‐terminal portion) and at least seven of the nine transmembrane domains is expressed resulting in defective function (Luuk et al. 2008; Koks et al. 2009).

WFS1‐deficient pancreatic islets secreted less insulin after incubation in 2 mmol/L and 10 mmol/L glucose solution compared to WT and Wfs1HZ islets. Surprisingly the overall secretion after stimulation was not as high as expected and there was no statistically significant difference after stimulation with 20 mmol/L glucose, however, there was a trend that Wfs1KO islets secreted less insulin. The rise of insulin secreted after stimulation with 10 mmol/L compared to 20 mmol/L glucose was greater for Wfs1KO islets while the amount of secreted insulin stayed more or less at the same level for WT and Wfs1HZ islets, which was not expected and could not be explained. In general this result is in accordance with previous studies made by Ishihara et al. (2004) and Riggs et al. (2005), although Ishihara et al. (2004) could not see a difference in insulin secretion after incubation in 2.5 mmol/L glucose (Ishihara et al. 2004; Riggs et al. 2005). This variation could be due to differences in *Wfs1* mouse models as current WFS1‐deficient mice have a disrupted exon 8 (Koks et al. 2009) and the other model is a complete knock‐out of exon 2 (Ishihara et al. 2004). The disruption of exon 8 leads to a dysfunctional protein mimicking more accurately Wolfram syndrome as most mutations in the *WFS1* gene occur in exon 8 (Cryns et al. 2003). Wfs1KO islets also showed a significant reduction in the amount of insulin secreted in response to sulfonylurea tolbutamide. Tolbutamide forces *β*‐cells to secrete insulin independent of glucose metabolism by closing the ATP‐sensitive K^+^ (K_ATP_) channels directly which results in membrane depolarization, Ca^2+^ influx and insulin release (Ashcroft and Rorsman 1989). WFS1 may therefore exert its effect on insulin secretion at or downstream of the K_ATP_ channel.

In vivo studies with WFS1‐deficient mice have shown that Wfs1KO mice have lower plasma insulin level than WT mice (Noormets et al. 2011; Terasmaa et al. 2011). In this study, there was no difference in insulin secretion between WT and Wfs1HZ, which is in accordance with in vivo results by Terasmaa et al. (2011). The lower insulin amount seen in Wfs1KO mice was primarily due to decreased insulin secretion, because the insulin content in pancreatic islets did not significantly differ between genotypes. These and previous results indicate a defect in insulin secretion in WFS1‐deficient pancreatic *β*‐cells (Ishihara et al. 2004; Hatanaka et al. 2011).

Hatanaka et al. (2011) showed that WFS1 localized in *β*‐cells and also to secretory granules and WFS1 deficiency caused changes in the intragranular pH, which in turn caused defects in proinsulin processing. Therefore, the level of circulating proinsulin is higher in WFS1‐deficient mice (Noormets et al. 2011). In this study, there were no significant differences in proinsulin amount per pancreatic islet between genotypes, although there was a trend that Wfs1KO had an increased level of proinsulin. The Wfs1KO proinsulin/insulin ratio was significantly higher compared to WT and Wfs1HZ only after incubation in 2 mmol/L glucose solution. There was also a trend that Wfs1KO proinsulin/insulin ratio was increased after stimulation with other solutions. Higher proinsulin/insulin ratio is associated with T2D (Mykkanen et al. 1997) indicating that WFS1‐deficient mice have a diabetic phenotype. The current results also indicate that there may be a problem with conversion of proinsulin to insulin and release of insulin from granules. This is in accordance with previous studies by Hatanaka et al. (2011).

According to functional annotation of RNA‐seq results genes with highest expressional changes due to WFS1 deficiency belong to the “tissue morphology, endocrine system development and function, molecular transport” functional network. Although only four samples of each genotype were sequenced, RNA sequencing revealed that melastatin‐related transient receptor potential subfamily member 5 (*Trpm5*) is downregulated in that pathway of Wfs1KO pancreatic islets. TRPM5 (melastatin‐related transient receptor potential subfamily member 5 protein) is expressed in the pancreatic islets of Langerhans and regulates Ca^2+^ oscillations contributing to insulin secretion (Brixel et al. 2010; Colsoul et al. 2010). Colsoul et al. (2010) showed that the action potentials of *Trpm5*‐mutant islet cells did not change, but the frequency of oscillations did. *Trpm5*‐mutant islet cells maintain slow oscillations but lack of fast oscillations (Colsoul et al. 2010).

TRPM5 is shown to regulate glucose‐stimulated insulin secretion as *Trpm5* knock‐out mice have impaired glucose tolerance. They have prolonged elevation of blood glucose levels, suggesting disturbances in insulin secretion (Brixel et al. 2010; Colsoul et al. 2010). TRPM5 forms a functional calcium‐activated nonselective cation channel conducting mainly Na^+^ and K^+^ ions without significant permeation to Ca^2+^. Its activation presumably causes membrane depolarization downstream of the closure of K_ATP_ channels (Prawitt et al. 2003; Brixel et al. 2010), what may be an important step leading to activation of voltage‐dependent calcium channels in glucose stimulated insulin secretion (Henquin et al. 2009; Brixel et al. 2010). Brixel et al. (2010) hypothesize that TRPM5 may also have an additional role in the vesicle–membrane fusion process as *Trpm5‐*mutant islets had still decreased insulin secretion when stimulated with arginine (Brixel et al. 2010). Although the exact mechanisms how TRPM5 activity is regulated and how it combines different types of sensory input in different cells are not known, the proposed hypothesis would help to explain how TRPM5 and WFS1 might interact or influence each other. The functional annotation of RNA‐seq results also showed that WFS1 and TRPM5 might be connected over proinsulin processing and/or Ca^2+^ signaling as it has been reported that the islets of *Wfs1* knock‐out mice have impaired Ca^2+^ signaling (Ishihara et al. 2004).

Further studies are needed to verify the functional interaction between WFS1 and TRPM5 in the regulation of insulin secretion and how this downregulation may contribute to diabetes‐like phenotype of WFS1‐deficient mice. Although this study has some limitations, mainly regarding islet stimulation induction and sample size, in conclusion, mice with disrupted *Wfs1* gene had fewer pancreatic islets and defective insulin secretion explaining their diabetes‐like phenotype. RNA‐sequencing of pancreatic islets showed that interestingly *Trpm5* is downregulated in WFS1‐deficient islets and the pathways related to tissue morphology, endocrine system development and function, molecular transport network are influenced.

## Conflict of Interest

No conflicts of interest, financial or otherwise, are declared by the author(s).
